# Understanding possible differences between haemodialysis and haemodiafiltration which affect survival in end-stage kidney disease dialysis patients: the STITCHED study halted by the COVID-19 epidemic

**DOI:** 10.3310/nihropenres.14213.2

**Published:** 2026-08-04

**Authors:** Andrew Davenport, Stephanie MacNeil, Fergus Caskey

**Affiliations:** 1 UCL Centre for Kidney & Bladder Health, University College London Medical School, London, England, NW3 2PF, UK; 2 Bristol Trials Centre, University of Bristol Medical School, Bristol, England, BS8 1NU, UK; 3 UK Population Health Sciences, University of Bristol Medical School, Bristol, England, BS8 2PS, UK

**Keywords:** β2 microglobulin, cognitive function, echocardiography, haemodiafiltration, haemodialysis, hypotension, pulse wave velocity

## Abstract

**Background:**

The mortality rate of kidney dialysis patients remains high, predominantly due to cardiovascular disease. The clearance of uraemic solutes with standard high-flux haemodialysis (HD) is mainly mediated by diffusion. More recently, haemodiafiltration (HDF), which adds convection, thus increasing the spectrum of cleared uraemic toxins, has been introduced into clinical practice. Recent reports have suggested that HDF treatment improves patient outcomes. However, it is unclear as to the amount of HDF treatment required or why HDF should improve patient outcomes.

The UK High-Volume Haemodiafiltration vs. High-Flux Haemodialysis Registry Trial (H4RT) study compared a composite of non-cancer mortality or hospital admission with a cardiovascular event or infection over 3 years. However, this study was not designed to investigate why HDF may have improved patient outcomes; therefore, the STITCHED study (Study To Investigate The Change in Hypotensive Episodes during Dialysis) was designed to be nested within the larger H4RT study.

**Objective:**

The STITCHED study was designed to investigate the potential mechanisms underlying the improved survival reported with HDF.

**Design and methods:**

The STITCHED study was designed to investigate changes in intra- and inter-dialytic blood pressure; differences in the clearance of uraemic toxins, temperature, sodium, and calcium balance between HDF and HD treatments; and the effects on the heart and brain by measuring differences in arterial stiffness, cardiac dimensions, and cognitive function.

**Setting and participants:**

Nine acute hospital trusts across England were contacted and involved in the STITCHED study. However, after delays due to covid-19 only the Royal Free Hospital in London recruited participants.

**Results:**

After disruptions due to the covid-19 pandemic the study was stopped before any results were obtained.

**Conclusion:**

The STITCHED study was halted before any relevant information was obtained, and as such was unable to answer the question of why one mode of dialysis should improve patient survival.

## Plain Language summary

**Background:**

Kidney failure patients treated by haemodialysis are at greater risk of stroke and vascular dementia. Blood pressure often falls during a kidney dialysis treatment and this is thought to cause damage to the brain. A different type of kidney dialysis, haemodiafiltration, is reported to cause fewer drops in blood pressure during treatment and removes a wider range of toxins from the blood.

**Aims and objectives:**

We wanted to investigate whether these early reports equally apply to UK dialysis patients. As to whether haemodiafiltration causes fewer falls in blood pressure during treatment and if this then leads to better preservation of cognitive function and fewer effects on the heart. We also wanted to know whether improved outcomes for patients were due to better blood pressure control or to the removal of different toxins by the different types of kidney dialysis treatments.

**Methods:**

We planned to record blood pressure both during dialysis sessions and between sessions using a wearable blood pressure machine, and look at changes to the heart with an echocardiogram. In addition, investigate whether the two types of dialysis treatment had different effects on cleaning the blood during a dialysis session by removing different toxins.

**Key findings:**

The study was halted before there were any findings.

**Conclusions and future plans:**

STITCHED was designed to fill a gap in knowledge about why patients treated with haemodiafiltration might have better survival than those treated with high-flux haemodialysis. Knowing why there should be a difference would potentially then lead to changes in the design of kidney dialysis machines, manufacturing of kidney dialysers, choice of dialysis fluids and changes in targets for blood pressure management. As STITCHED was halted this knowledge gap remains.

## Background

The mortality of end-stage kidney dialysis patients remains high, with the 5-year survival rate being lower than that of more common solid organ cancers. Cardiovascular diseases are the most common cause of mortality, followed by infections. In addition to a 10-fold increase in the risk of stroke, elderly dialysis patients who start dialysis are reported to have an increased risk of progressive vascular dementia. Standard dialysis treatments are based on the diffusive clearance of uraemic toxins, with high-flux haemodialysis (HD) being the current standard of treatment. Recently, online haemodiafiltration (OL-HDF) has been introduced into clinical practice. This adds convective clearance, thus increasing the spectrum of uraemic solutes that can be removed from the blood. However, three major clinical trials were completed in 2017/2018 and failed to show a difference in patient outcomes between these two dialysis modalities. However, these trials did not target a specific amount of convective clearance. Another trial targeting a high dose of convective clearance reported better patient survival with high-volume OL-HDF. To investigate the amount of convective clearance required to improve patient outcomes, the UK High-Volume Haemodiafiltration vs. High-Flux Haemodialysis Registry Trial (H4RT) study to compare high-volume OL-HDF with standard HD was proposed.

The HR4T trial enrolled end-stage kidney dialysis patients in the UK into a randomised prospective trial comparing high-volume OL-HDF and HD treatments. ^1^ The trial collected data on patient demographics, comorbidities, and medications. Longitudinal laboratory results and dialysis session data were also collected by linking to the UK Renal Registry. The H4RT study endpoints included a comparison of hospital admissions due to cardiovascular and infection-related events, patient survival and mortality data, patient-reported outcomes, and health care economics. The primary outcome was a composite of non-cancer mortality or hospital admissions with a cardiovascular event or infection during a 3-year follow-up period.

## Study design

The H4RT study was designed to investigate the amount of convective clearance required to improve patient outcomes. However, it was not set up to determine why HDF could provide improved patient outcomes. The STITCHED study (Study To Investigate the Change in Hypotensive Episodes during Dialysis) was designed as a mechanistic study nested within the main H4RT study. Recruiting patients already entered into the H4RT study, randomised to either standard high-flux HD or high-volume OL-HDF, and followed for 2 years.

Previous studies comparing high-flux HD and OL-HDF would suggest that mortality, particularly cardiovascular mortality and stroke, is more likely to be reduced with OL-HDF. ^2– 8^ The H4RT study was designed to deliver high-volume OL-HDF convective exchanges, and these greater exchange volumes are expected to increase the thermal losses during treatments. Previous studies have demonstrated that negative thermal balance increases sympathetic nervous system activity and reduces the risk of intra-dialytic hypotension. ^9– 11^ Fewer hypotensive episodes during treatment coupled with more stable systolic blood pressure control would be expected to reduce intradialytic hypotension and potential ischaemic damage to the heart and brain. A more stable blood pressure would also allow more reliable fluid removal, so by reducing the risks of volume overload and leading to reduced patient morbidity and mortality. ^12– 15^ Thus, one possible mechanism that may explain the potential benefit of OL-HDF is more stable blood pressure. The European Dialysis and Transplant Association recommended ambulatory blood pressure monitoring for patients with end-stage kidney disease treated with dialysis. ^16^ As such, we planned to monitor the blood pressure during both dialysis sessions and the interval between dialysis sessions. Additionally, both observational data and experimental evidence suggest that OL-HDF may reduce or prevent an increase in arterial vascular stiffness. Advances in ambulatory blood pressure monitoring also permit the measurement of vascular stiffness using pulse wave velocity. ^17^ Thus, we planned to determine whether there was any improvement or reduction in vascular stiffness over time with OL-HDF compared with standard HD. As some of the uraemic toxins that accumulate in patients with end-stage kidney disease have been implicated in reducing arterial compliance and reactivity, we planned to determine whether there are changes in uraemic toxins with OL-HDF treatments.

The risk of stroke, particularly ischaemic stroke, has been reported to increase in end-stage kidney dialysis patients. Similarly, elderly HD patients have been reported to be at a greater risk of developing vascular dementia. As episodes of intradialytic hypotension are reported to reduce cerebral blood flow during dialysis sessions, we planned to assess whether differences in blood pressure stability between HD and OL-HDF led to differences in cognitive function over time using the Montreal Cognitive Assessment (MoCA) test. ^18^ Similarly, dialysis patients gain fluid between dialysis sessions. Hypotensive episodes during dialysis stop fluid removal, thus increasing the risk of patients leaving the dialysis volume overloaded. To investigate whether more stable intradialytic blood pressure with OL-HDF has beneficial effects on the heart, we were to use echocardiography to assess structural and functional changes ^19, 20^ and measure volume status with bioelectrical impedance measurements. ^21, 22^

## Study objectives ^23^

**Primary objective:**

a) To compare intradialytic hypotensive episodes between high flux haemodialysis (HD) and online haemodiafiltration (OL-HDF).

**Secondary objectives:**

a) To determine whether there are differences in blood pressure and blood pressure variability over time between high-flux HD and OL-HDF.
b) To determine whether there are differences in pulse wave velocity over time between high-flux HD and OL-HDF.
c) To determine whether there are differences in changes in cognitive function between high-flux HD and OL-HDF.
d) To determine whether there are differences in extracellular water volumes between high-flux HD and OL-HDF.
e) To determine whether there are differences in changes in cardiac biomarkers between high-flux HD and OL-HDF.
f ) To determine whether there are changes in cardiac echocardiographic parameters and function between high-flux HD and OL-HDF.
g) To determine whether there are differences in the clearance of the middle molecule β2 microglobulin between high-flux HD and OL-HDF.

## Ethics

We planned to recruit adult kidney dialysis patients who had provided written informed consent and were enrolled into the H4RT trial. These patients were then given STITCHED study information and subsequently then asked to provide written informed consent to then be recruited into the STITCHED sub-study. In keeping with the Helsinki accord only patients who could provide written informed consent were recruited into STITCHED. The STITCHED study was approved by the East Midlands – Leicester Central Research Ethics committee 18/EM/0212.

## Study methods

We planned to capture the following data, had the study not been halted.

### Demographics

Patients who had provided written informed consent and enrolled in the main H4RT study had demographic, medical history, medication prescriptions, and comorbidity data collected by research nurses at the H4RT study entry.

It was planned that this data would be collected as part of H4RT, and then shared with STITCHED for patients who were co-enrolled in both studies.

In addition, patients who entered the STITCHED were asked to complete patient-reported symptoms at study entry and then at 6 monthly intervals. Follow-up data were collected by H4RT from the UK Renal Registry and Hospital Episode Statistics (HES), which would then be made available for STITCHED.

### Laboratory data

**Blood testing**

5 mL blood samples would be taken and stored for subsequent analysis of cardiac biomarkers on three occasions, at study entry, 12 months and on study completion at 24 months. Samples were taken prior to dialysis when other blood samples were taken as part of routine clinical management.

5 mL blood samples would be taken pre- and post the dialysis session and stored for subsequent analysis of β2 microglobulin on three occasions, at study entry, 12 months and on study completion at 24 months. Samples were taken when other blood samples were taken as part of routine clinical management and stored for analysis of biomarkers and uraemic toxins.

### Blood pressure and pulse wave velocity

**Intra-dialytic hypotension**

The UK Renal Association advocates that dialysis centres record episodes of intradialytic hypotension as a part of good clinical practice. Research nurses collect monthly data from electronic dialysis records or in cases of centres that do not have a computerised electronic record from paper dialysis sheet records of intradialytic hypotension, defined by the European Best Practice Clinical Guidelines, and the severity of episodes (ultrafiltration stopped or administration of intravenous fluids). ^11^

**Inter-dialytic blood pressure monitoring**

Patients were requested to wear ambulatory blood pressure devices during the first dialysis session of the week and then during the interval to the subsequent dialysis session, at study entry, at 12 months, and at study completion at 24 months. Advances in ambulatory monitoring now allow lighter, more comfortable devices to be used with greater patient satisfaction and also allow measurement of vascular stiffness by pulse wave velocity.

Analysis of ambulatory blood pressure measurements was performed centrally at the Department of Cardiology, Royal Free Hospital.

### Echocardiography

Patients were requested to undergo transthoracic echocardiography on a non-dialysis day, at study entry, and after 24 months to determine changes in cardiac anatomy and assess systolic and diastolic function. Echocardiogram images were sent after anonymisation via an encrypted NHS net to the cardiology department at the Royal Free Hospital for central analysis.

### Cognitive function

The Montreal Cognitive Assessment (MoCA) is a tool used by many healthcare professionals working in the NHS to assess cognitive function. Research nurses were instructed to administer the MoCA to patients during the first hour of dialysis treatment during the mid-week dialysis session at study entry, 12 months, and 24 months at the end of the study.

### Bioimpedance

Changes in extracellular water and intracellular water were assessed by multifrequency bioimpedance at study entry, 12 months, and 24 months after study conclusion. Measurements were made by research study nurses and standardised prior to the midweek dialysis session.

### Study recruitment – Sample size

Sample sizes were calculated by the Bristol Randomised Trials Collaboration (BRTC) using the most recent trial of higher-volume HDF compared with high-flux haemodialysis. Assuming a clinically important effect of reducing intradialytic hypotension by 30% with high-volume OL-HDF, 138 patients recruited into each arm (276 in total) would be required for 90% power at the 5% significance level. This would be achieved by six centres recruiting four patients per month for 12 months, giving a total of 288 patients. The trial was designed as a pilot, to highlight which factor(s) could have impacted most on improved patient survival, so then to form the basis of a future interventional trial.

**Study Registration Number**: IRAS 236894 and R&D 17/0869

Ethical committee approval Leicester Central 18/EM/0212

Trial registration number ISRCTN74893205

UCL data protection registration number Z6364106/2018/04/135

Zenodo repository 10.5281/zenodo20154880

## Equality, diversity and inclusion

Patient groups included the patient advisory board for the UK Renal Registry and the Royal Free Hospital Kidney Patients’ Association. These committees comprised patients of different sexes, religions, and ethnicities. Patient recruitment at the main site, the Royal Free Hospital, included patients of different sexes, religions, and ethnic backgrounds.

## Patient and public involvement in the study

### Aim

The study was approved by the patient representative committee at the Renal Registry and local Royal Free Hospital Kidney Patients’ Association. Advice was given in both drawing up consent and patient information forms. The study protocol was also discussed in terms of how many blood tests should be performed, as well as the number and frequency of additional investigations, including ambulatory blood pressure monitoring, bioimpedance measurements, and echocardiography examinations.

### Methods

Patient Committees were sent documents for review, and a face-to-face discussion was held with the local Royal Free Hospital Kidney Patients’ Association committee.

### Study results

Following advice from patient representative committees, changes were made in the wording of both patient study information and consent forms. As the study was stopped prematurely, there were no results to be discussed. University College London, as the sponsor, has a series of formalised study forms, as some of the patient representatives wished to make changes to the structure of these forms, as they found some sections of the forms difficult to understand and queried the relevance to the current study.

### Discussion and conclusions

As the study was prematurely stopped there were no results to be discussed.

### Reflective/critical perspective

The involvement of patient representatives was useful in determining the number of blood tests to be performed, as well as the number and frequency of additional investigations, including ambulatory blood pressure monitoring, bioimpedance measurements, and echocardiography examinations.

## Results

The study was halted before any relevant data was obtained and as such statistical analysis could not be undertaken.

## Discussion

The study was halted before any relevant findings were obtained due to the withdrawal of funding from the NIHR. The study began in the summer of 2020, immediately after COVID-19 reached London. The Royal Free Hospital London was the primary site for STITCHED, and the hospital initially stopped all clinical research. This meant that the research team could not visit kidney dialysis centres, and consequently, all patient recruitment was halted. This practice of closing down clinical research activities was mirrored in our other participating centres. Our renal research team was seconded to other posts within the hospital or to COVID-19 studies. When clinical research resumed, COVID-19 and cancer studies were prioritised. Research activities were restarted in a stepwise manner, with renal research in the last tier to be reactivated. We and our other centres that had agreed to participate in STITCHED found that when we were allowed to recommence renal clinical research studies, we had fewer renal research staff. The reduction in staffing was due to a combination of staff leaving or moving to other posts, as there had been no clinical renal research, long-term staff sickness due to COVID-19, and no new staff recruitment to replace the staff members who had left.

As STITCHED fell behind in the study milestones, a review was conducted by the NIHR. A number of problems were highlighted and solutions proposed by the review committee, which included more local ownership of the development of STITCHED-specific clinical research forms. The University College London surgical trial group was subcontracted to produce and test a new series of study case forms. The costs for this work were paid independently from internal sources and not from the STITCHED monies. New milestone targets were set by the trial review committee, but despite meeting these targets, the NIHR decided to withdraw funding for the STITCHED study, and the study was halted.

When funding was withdrawn for STITCHED, 98 patients had consented to participate in the study. As requested in the study closure notification, all blood samples which had been collected for batch analysis were destroyed and not analysed. Only a minority of patients had completed baseline ambulatory blood pressure and pulse wave velocity measurements at that time, in keeping with directions for study closure any preliminary data was destroyed.

## Conclusion and future directions

The STITCHED study was halted before meaningful results were obtained to draw any conclusions. The H4RT study subsequently completed recruitment and is currently in the final analysis stage. Recently, a European trial comparing high-volume OL-HDF and standard high-flux HD (CONVINCE) reported some of their initial findings, with higher survival for those treated with high-volume OL-HDF. ^24^ Although both trials appear to compare the same dialysis modes, there are differences in patient recruitment, target convective exchange volumes, and primary outcomes. However, neither trial was designed to answer the question: “if high volume OL-HDF does improve survival for kidney dialysis patients, what is the reason?” This was the rationale behind the STITCHED study.

The STITCHED study was designed to compare changes in blood pressure during dialysis treatments, the inter-dialytic interval, and the clearance of uraemic toxins and biomarkers. By comparing these changes in arterial stiffness, cardiac modelling, and cognitive function, STITCHED was designed to determine whether the improvement in survival was due to improved blood pressure control, removal of uraemic toxins, and changes in the thermal or sodium and calcium balance between diffusive and convective clearance. The reason for improved survival is important in deciding the future of dialysis treatments. For example, there is now a new design of dialysers that allows for a similar spectrum of uraemic toxins to be removed by internal convection compared to HDF with greater clearance compared to diffusion.

The economic healthcare costs of high-volume OL-HDF compared to high-flux haemodialysis were reported to be greater in the CONVINCE study group. Although this was partly due to longer patient survival, the sessional cost of an individual dialysis treatment was greater with OL-HDF; however, this cost was considered acceptable when considering the benefits to patient healthcare. Although dialysis machines for OL-HDF require three pumps, a conventional haemodialysis machine for diffusive clearance only requires two pumps; therefore, it is less complex, has fewer parts, and may potentially have lower capital and running costs. On the other hand, if survival is related to improved blood pressure control during treatment, this may reflect the differences in thermal balance between convective and diffusive modes, and dialysers and dialysis machines need to be developed to maximise convective exchange so that all patients could benefit from high-volume OL-HDF. Again, if OL-HDF leads to better blood pressure control during the inter-dialytic interval, bioimpedance-measured volume control, and sodium and calcium balance between the different modalities, this may alter the choice and composition of dialysis fluids.

Although H4RT and CONVINCE should provide more evidence as to whether OL-HDF improves patient outcomes and is cost-effective compared to high-flux HD, an understanding of the mechanisms underlying these outcomes will remain unexplored without further research.

## Role of the sponsor

The sponsor provided funding for the study but did not materially influence the conduct of the study until it was closed down, or influence any interpretation of study findings.

## Data sharing statement

As the study was stopped prematurely, no significant data were collected for sharing. Data protection was provided by the UCL, according to the GDPR guidelines.

## Disclaimer

This study was funded by the National Institute for Health and Care Research (NIHR) under the EME program (NETSCC 17/61/04). The views expressed are those of the author and not necessarily those of the NIHR or Department of Health and Social Care. This project was carried out between May 2019, when regulatory approval was obtained, and November 2021. The program examined the report at the time of submission to assess completeness against the stated aims. These reports did not involve a peer-review process https://doi.org/10.1186/ISRCTN74893205.

## Data Availability

As the study was stopped prematurely, before any STITCHED study data was collected then there is no data available for sharing ( 10.5281/zenodo20154880). ^23^

## References

[1] Caskey FJ Procter S MacNeill SJ : The high-volume haemodiafiltration vs high-flux haemodialysis registry trial (H4RT): a multi-centre, unblinded, randomised, parallel-group, superiority study to compare the effectiveness and cost-effectiveness of high-volume haemodiafiltration and high-flux haemodialysis in people with kidney failure on maintenance dialysis using linkage to routine healthcare databases for outcomes. *Trials.* 2022 Jun 27;23(1):532. 10.1186/s13063-022-06357-y 35761367 PMC9235280

[2] Ok E Asci G Toz H : Mortality and cardiovascular events in online haemodiafiltration (OL-HDF) compared with high-flux dialysis: results from the Turkish OL-HDF Study. *Nephrol. Dial. Transplant.* 2013;28(1):192–202. 23229932 10.1093/ndt/gfs407

[3] Grooteman MP Dorpel MA van den Bots ML : Effect of online hemodiafiltration on all-cause mortality and cardiovascular outcomes. *J. Am. Soc. Nephrol.* 2012;23(6):1087–1096. 10.1681/ASN.2011121140 22539829 PMC3358764

[4] Morena M Jaussent A Chalabi L : Treatment tolerance and patient-reported outcomes favor online hemodiafiltration compared to high-flux hemodialysis in the elderly. *Kidney Int.* 2017;91(6):1495–1509. 10.1016/j.kint.2017.01.013 28318624

[5] Peters SA Bots ML Canaud B : HDF Pooling Project Investigators. Haemodiafiltration and mortality in end-stage kidney disease patients: a pooled individual participant data analysis from four randomized controlled trials. *Nephrol. Dial. Transplant.* 2016;31(6):978–984. 26492924 10.1093/ndt/gfv349

[6] Davenport A Peters SA Bots ML : HDF Pooling Project Investigators. Higher convection volume exchange with online hemodiafiltration is associated with survival advantage for dialysis patients: the effect of adjustment for body size. *Kidney Int.* 2016;89(1):193–199. 10.1038/ki.2015.264 26352299

[7] Nubé MJ Peters SA Blankestijn PJ : Mortality reduction by post-dilution online-haemodiafiltration: a cause-specific analysis. *Nephrol. Dial. Transplant.* 2016 Dec 26;32:548–555. 10.1093/ndt/gfw381 28025382

[8] Maduell F Moreso F Pons M : High-efficiency post-dilution online hemodiafiltration reduces all-cause mortality in hemodialysis patients. *J. Am. Soc. Nephrol.* 2013;24(3):487–497. 10.1681/ASN.2012080875 23411788 PMC3582206

[9] Davenport A : Intradialytic complications during haemodialysis. *Hemodial. Int.* 2006;10(2):162–167. 10.1111/j.1542-4758.2006.00088.x 16623668

[10] Flythe JE Xue H Lynch KE : Association of mortality risk with various definitions of intradialytic hypotension. *J. Am. Soc. Nephrol.* 2015;26(3):724–734. 10.1681/ASN.2014020222 25270068 PMC4341481

[11] Kooman J Basci A Pizzarelli F : EBPG guideline on haemodynamic instability. *Nephrol. Dial. Transplant.* 2007;22:ii22–ii44. 10.1093/ndt/gfm019 17507425

[12] Beerenhout C Kooman JP Claessens P : Thermal effects of different dialysis techniques and blood pump speeds: an in vitro study. *J. Nephrol.* 2003;16(4):552–557. 14696758

[13] Daugirdas JT : Lower cardiovascular mortality with high-volume hemodiafiltration: a cool effect? *Nephrol. Dial. Transplant.* 2016 Jun;31(6):853–856. 10.1093/ndt/gfv412 26687900

[14] Eldehni MT Odudu A McIntyre CW : Randomized clinical trial of dialysate cooling and effects on brain white matter. *J. Am. Soc. Nephrol.* 2015;26(4):957–965. 10.1681/ASN.2013101086 25234925 PMC4378094

[15] Selby NM McIntyre CW : How should dialysis fluid be individualized for the chronic hemodialysis patient? *Temperature. Semin Dial.* 2008;21(3):229–231. 10.1111/j.1525-139X.2008.00429.x 18363597

[16] Parati G Ochoa JE Bilo G : Hypertension in Chronic Kidney Disease Part 2: Role of Ambulatory and Home Blood Pressure Monitoring for Assessing Alterations in Blood Pressure Variability and Blood Pressure Profiles. *Hypertension.* 2016 Jun;67(6):1102–1110. 10.1161/HYPERTENSIONAHA.115.06896 27141057

[17] Koutroumbas G Georgianos PI Sarafidis PA : Ambulatory aortic blood pressure, wave reflections and pulse wave velocity are elevated during the third in comparison to the second interdialytic day of the long interval in chronic haemodialysis patients. *Nephrol. Dial. Transplant.* 2015 Dec;30(12):2046–2053. 10.1093/ndt/gfv090 25920919 PMC4832990

[18] Kurella Tamura M Covinsky KE Chertow GM : Functional status of the elderly before and after dialysis initiation. *N. Engl. J. Med.* 2009 Oct 15;361(16):1539–1547. 10.1056/NEJMoa0904655 19828531 PMC2789552

[19] Kim ED Sozio SM Estrella MM : Cross-sectional association of volume, blood pressure, and aortic stiffness with left ventricular mass in incident haemodialysis patients: Predictors of Arrhythmic and Cardiovascular Risk in End-Stage Renal Disease (PACE) study. *BMC Nephrol.* 2015 Aug 7;16:131. 10.1186/s12882-015-0131-4 26249016 PMC4528691

[20] Canaud B Kooman JP Selby NM : Dialysis-induced cardiovascular and multiorgan morbidity. *Kidney Int. Rep.* 2020 Sep 9;5(11):1856–1869. 10.1016/j.ekir.2020.08.031 33163709 PMC7609914

[21] Davies SJ Davenport A : The role of bioimpedance and biomarkers in aiding clinical decision-making in volume assessments in dialysis patients. *Kidney Int.* 2014;86(3):489–496. 10.1038/ki.2014.207 24918155

[22] Onofriescu M Hogas S Voroneanu L : Bioimpedance-guided fluid management in maintenance hemodialysis: a pilot randomized controlled trial. *Am. J. Kidney Dis.* 2014;64(1):111–118. 10.1053/j.ajkd.2014.01.420 24583055

[23] Davenport A MacNeill S Caskey F : Study To Investigate The Change in Hypotensive Episodes during Dialysis (STITCHED) (version 2). *Zenodo repository.* 10.5281/zenodo20154880

[24] Blankestijn PJ Vernooij RWM Hockham C : Effect of hemodiafiltration or haemodialysis on mortality in kidney failure. N. Engl. J. Med. 2023 Aug 24;389(8):700–709. 37326323 10.1056/NEJMoa2304820

