# Supplementary material for: Understanding possible differences between haemodialysis and haemodiafiltration which affect survival in end-stage kidney disease dialysis patients: the STITCHED study halted by the COVID-19 epidemic
Source: NIHR Open Res. 2026 Aug 4;6:27. Originally published 2026 Mar 19. [Version 2] doi: 10.3310/nihropenres.14213.2 (PMC13563014; doi:10.3310/nihropenres.14213.2)
Supplement: Peer review history

# Reviewer report: Reviewer response for version 2

**DOI:** 10.3310/nihropenres.15640.r41561

**Published:** 2026-09-02

**Authors:** Saquiba Yesmine

**Affiliations:** 1 Jahangirnagar University, Savar, Dhaka Division, Bangladesh

I read with great interest the protocol and administrative outcomes of the STITCHED study entitled "Understanding possible differences between haemodialysis and haemodiafiltration which affect survival in end-stage kidney disease dialysis patients: The STITCHED Study halted by the COVID- 19 epidemic". The investigators should be commended for addressing a critical issue in end stage kidney disease patients. Previous randomized trials, including the CONVINCE trial, have shown a reduction in all-cause mortality with high-dose haemodiafiltration (HDF), the exact underlying physiological mechanisms remain unclear.

 The STITCHED protocol's strength is that it included comprehensive framework, assessing different factors such as stability of blood pressure, electrolyte balance, cardiac dimensions, and cognitive function, thus linking macro-level data to micro-level protection.

 However, the protocol was very complex and not very sturdy. It was depended on specialized testing across hospitals, thus any disruption in the supply chain or patient availability could cause it to fail to reach any clinical conclusions.

 The COVID-19 interruptions shrunken data collection. The limited recruitment at a single center and a premature study halt, caused a loss of statistical power. A gap in our understanding is created without actionable mechanistic data. Although, evidence suggests that high-volume HDF improves survival, however, data for why a survival benefit exists is still lacking.

 The STITCHED trial serves as a crucial blueprint for future trial designs, highlighting the need for multi-center mechanistic studies that can withstand disruptions.

 In summary, although the STITCHED study could not fulfill its primary endpoints, its scientific rationale remains valid. The study should be continued to multi-center mechanistic trials to help clinicians understand the precise physiological reasons behind convective survival benefits.

 Moreover, replication of the objectives of the STITCHED study using real-world target trial or multi-site physiological sub-studies is essential if we want to fully optimize and personalize convective therapies for end-stage kidney disease patients.

Is the work clearly and accurately presented and does it cite the current literature?

Yes

If applicable, is the statistical analysis and its interpretation appropriate?

Not applicable

Are all the source data underlying the results available to ensure full reproducibility?

No source data required

Is the study design appropriate and is the work technically sound?

Yes

Are the conclusions drawn adequately supported by the results?

Partly

Are sufficient details of methods and analysis provided to allow replication by others?

Partly

Reviewer Expertise:

Diabetes, Chronic kidney disease

I confirm that I have read this submission and believe that I have an appropriate level of expertise to confirm that it is of an acceptable scientific standard, however I have significant reservations, as outlined above.

## References

[1] : Effect of Hemodiafiltration or Hemodialysis on Mortality in Kidney Failure. *New England Journal of Medicine* .2023;389(8) : 10.1056/NEJMoa2304820700-709 10.1056/NEJMoa2304820 37326323

# Reviewer report: Reviewer response for version 2

**DOI:** 10.3310/nihropenres.15640.r41445

**Published:** 2026-08-31

**Authors:** Jeroen Vollenbroek, Robin Vernooij

**Affiliations:** 1 Nephrology, University Medical Centre Utrecht, Utrecht, The Netherlands

No further comments

Is the work clearly and accurately presented and does it cite the current literature?

No

If applicable, is the statistical analysis and its interpretation appropriate?

Not applicable

Are all the source data underlying the results available to ensure full reproducibility?

No

Is the study design appropriate and is the work technically sound?

Yes

Are the conclusions drawn adequately supported by the results?

No

Are sufficient details of methods and analysis provided to allow replication by others?

Partly

Reviewer Expertise:

Dialysis innovation, Dialysate regeneration, artificial organs

We confirm that we have read this submission and believe that we have an appropriate level of expertise to confirm that it is of an acceptable scientific standard.

# Reviewer report: Reviewer response for version 2

**DOI:** 10.3310/nihropenres.15640.r41446

**Published:** 2026-08-26

**Authors:** Jakub Ruszkowski

**Affiliations:** 1 Medical University of Gdańsk, Gdańsk, Poland

I thank the authors for their revisions and their constructive engagement with the review.

 The editorial inconsistencies have been resolved: the ethics committee reference now reads consistently (18/EM/0212), the "STITCHED" spelling in the Discussion is corrected, and the journal name has been added to reference 24.

 One item remains. The Zenodo DOI as it currently appears in the text (10.5281/zenodo20154880) does not work; adding the period — 10.5281/zenodo.20154880 — makes the link work, so it may be worth checking the version cited in the manuscript body, reference 23, and the data-availability statement. Relatedly, the second author's surname still alternates between "MacNeil" and "MacNeill" in one or two places.

 On the reflective/critical perspective (previous comment 3): I understand the constraints, and the account already given in the Discussion is valuable. Should the authors ever feel able to add even a few sentences on what safeguards a nested mechanistic sub-study might need when an external shock (like COVID-19) disrupts a host trial, I suspect it would become one of the most widely read parts of the paper — offered as an invitation for a future version, not a requirement.

Is the work clearly and accurately presented and does it cite the current literature?

Partly

If applicable, is the statistical analysis and its interpretation appropriate?

Not applicable

Are all the source data underlying the results available to ensure full reproducibility?

No source data required

Is the study design appropriate and is the work technically sound?

Yes

Are the conclusions drawn adequately supported by the results?

Yes

Are sufficient details of methods and analysis provided to allow replication by others?

Yes

Reviewer Expertise:

Nephrology

I confirm that I have read this submission and believe that I have an appropriate level of expertise to confirm that it is of an acceptable scientific standard, however I have significant reservations, as outlined above.

# Reviewer report: Reviewer response for version 1

**DOI:** 10.3310/nihropenres.15474.r40305

**Published:** 2026-07-02

**Authors:** Guido Gembillo

**Affiliations:** 1 University of Messina, Messina, Italy

The manuscript addresses a genuinely important and under-explored question. Neither H4RT nor CONVINCE was designed to answer *why* high-volume OL-HDF might improve survival, and this mechanistic gap has direct implications for dialyser design, machine architecture, dialysate composition, and blood-pressure targets. A study that set out to address this gap, even one halted before completion, has documentary and scientific value.

 Several strengths deserve explicit recognition. The pathophysiological rationale is well articulated across the thermal-haemodynamic axis, blood-pressure variability, middle-molecule clearance, and hypoperfusion-related cerebral injury. The proposed measurement battery (ABPM with PWV, MoCA, echocardiography, bioimpedance, cardiac biomarkers, β2-microglobulin) is clinically appropriate and operationally realistic. Patient involvement through the Renal Registry advisory board and the Royal Free Kidney Patients' Association is exemplary. Transparent publication of a halted NIHR-funded study is itself a contribution to research integrity; it pulls the work out of the file drawer where most halted studies remain.

**Proposed revisions**

**1. Realign title and genre with the actual content.** The manuscript functions as a hybrid protocol-and-closure report, a revised title along the lines of *"The STITCHED study: protocol and closure report of a mechanistic sub-study of H4RT"*, paired with one explicit framing sentence at the end of the Background, will orient readers correctly and resolve the principal concern raised by both reviewers.

**2. State the position on the link between mediators and survival outcome.** The current objectives are pairwise HD-vs-OL-HDF comparisons, the narrative speaks of *explaining* survival. A short Methods sub-section, *"Statistical analysis plan and link to H4RT outcomes"*, should declare which framework applies. If a pre-closure SAP included formal mediation analysis (modality as exposure, STITCHED parameters as candidate mediators, H4RT survival and cardiovascular events as outcomes), summaries it in five to eight lines: framework type (causal mediation, counterfactual), handling of correlated mediators, planned software. If STITCHED was conceived as descriptive/hypothesis-generating with mediation deferred to a successor study, state this directly and sketch the successor design in the Conclusion (mediation-driven sample size, biologically prioritised mediators, prospective integration with H4RT/CONVINCE outcomes).

**3. Expand the reflective section, the manuscript's most distinctive potential contribution.** The most instructive material, pandemic disruption, research-staff attrition, the NIHR review process, the CRF subcontracting arrangement, funding withdrawal despite revised milestones being met, currently sits in the Discussion, while *"Reflective/critical perspective"* is nearly empty. Relocating and expanding this content, with generalisable lessons attached, will give the paper lasting value. Concrete questions to address: what contractual safeguards would protect a mechanistic sub-study nested within a larger trial during an external shock? What milestone flexibility from funders would be appropriate? How should sponsor-substudy relationships be structured to preserve continuity of funding? A well-developed reflective section will become the most-cited part of the paper and a genuine resource for anyone designing mechanistic sub-studies in the post-CONVINCE era.

**4. Report the data that were collected with greater granularity.** A short descriptive paragraph should report: number of the randomised patients within the sub-study (with arm allocation, if known), number of completed baseline ABPM, PWV, MoCA, BIA, and echocardiographic assessments, and explicit confirmation of the fate of the blood samples (destroyed on closure request). Even with numbers too small for inference, descriptive reporting strengthens the closure report's credibility.

**5. Reconcile the sample-size figures.** The discrepancy between the 288 calculated in the manuscript and the 346 registered in the trial registry deserves one explanatory sentence in the *Sample size* section, most likely drop-out inflation, mid-study revision, or conservative registry registration.

**6. Correct editorial inconsistencies.** Unify the ethics code (18/EM/0213 vs 18/EM/0212), the second author's surname (McNeil vs MacNeill), correct "SITCHED" → "STITCHED" in the Discussion, add *N Engl J Med* to reference 24 (Blankestijn 2023, CONVINCE), and verify the Zenodo DOI (10.5281/zenodo18526386), which is currently non-functional and is self-cited as reference 23.

**7. Optional update on H4RT.** Given that H4RT is described as "in final analysis stage" and the OL-HDF landscape is moving quickly, a brief status update, or incorporation of any preliminary findings available by final submission will increase the manuscript's temporal relevance.

 Once these revisions have been implemented, the manuscript will be suitable for publication and will address a genuine unmet need within the field. Halted, partial, and null-result studies are infrequently disseminated in the nephrology literature, and this under-representation perpetuates publication bias and obliges successive investigators to rediscover operational lessons that have already been learned at considerable cost .

Is the work clearly and accurately presented and does it cite the current literature?

Yes

If applicable, is the statistical analysis and its interpretation appropriate?

Partly

Are all the source data underlying the results available to ensure full reproducibility?

Yes

Is the study design appropriate and is the work technically sound?

Yes

Are the conclusions drawn adequately supported by the results?

Yes

Are sufficient details of methods and analysis provided to allow replication by others?

Partly

Reviewer Expertise:

My main areas of expertise are haemodialysis, biomarkers, clinical trial design, online haemodiafiltration, and acute dialysis.

I confirm that I have read this submission and believe that I have an appropriate level of expertise to confirm that it is of an acceptable scientific standard, however I have significant reservations, as outlined above.

# Response

**Published:** 2026-07-07

**Authors:** Andrew Davenport

Several strengths deserve explicit recognition. The pathophysiological rationale is well articulated across the thermal-haemodynamic axis, blood-pressure variability, middle-molecule clearance, and hypoperfusion-related cerebral injury. The proposed measurement battery (ABPM with PWV, MoCA, echocardiography, bioimpedance, cardiac biomarkers, β2-microglobulin) is clinically appropriate and operationally realistic. Patient involvement through the Renal Registry advisory board and the Royal Free Kidney Patients' Association is exemplary. Transparent publication of a halted NIHR-funded study is itself a contribution to research integrity; it pulls the work out of the file drawer where most halted studies remain.

**Proposed revisions**

**1. Realign title and genre with the actual content.** The manuscript functions as a hybrid protocol-and-closure report, a revised title along the lines of *"The STITCHED study: protocol and closure report of a mechanistic sub-study of H4RT"*, paired with one explicit framing sentence at the end of the Background, will orient readers correctly and resolve the principal concern raised by both reviewers.

 The title was revised in a previous version, , which unfortunately could not be uploaded into the website by the F1000 team

**2. State the position on the link between mediators and survival outcome.** The current objectives are pairwise HD-vs-OL-HDF comparisons, the narrative speaks of *explaining* survival. A short Methods sub-section, *"Statistical analysis plan and link to H4RT outcomes"*, should declare which framework applies. If a pre-closure SAP included formal mediation analysis (modality as exposure, STITCHED parameters as candidate mediators, H4RT survival and cardiovascular events as outcomes), summaries it in five to eight lines: framework type (causal mediation, counterfactual), handling of correlated mediators, planned software. If STITCHED was conceived as descriptive/hypothesis-generating with mediation deferred to a successor study, state this directly and sketch the successor design in the Conclusion (mediation-driven sample size, biologically prioritised mediators, prospective integration with H4RT/CONVINCE outcomes).

 This was amended in a previous version, which unfortunately could not be uploaded into the website by the F1000 team

**3. Expand the reflective section, the manuscript's most distinctive potential contribution.** The most instructive material, pandemic disruption, research-staff attrition, the NIHR review process, the CRF subcontracting arrangement, funding withdrawal despite revised milestones being met, currently sits in the Discussion, while *"Reflective/critical perspective"* is nearly empty. Relocating and expanding this content, with generalisable lessons attached, will give the paper lasting value. Concrete questions to address: what contractual safeguards would protect a mechanistic sub-study nested within a larger trial during an external shock? What milestone flexibility from funders would be appropriate? How should sponsor-substudy relationships be structured to preserve continuity of funding? A well-developed reflective section will become the most-cited part of the paper and a genuine resource for anyone designing mechanistic sub-studies in the post-CONVINCE era.

 Unless one had worked in London during the 2 main waves of the COVID-19 it is difficult for someone to understand the issues that arose. There is a current UK government enquiry into the preparedness and the response to the COVID pandemic. In essence all clinical research was halted as clinical, and non0clinical staff were re-deployed to support the acute clinical service. Subsequently COVID research was then permitted, and over time in a step wise fashion other research projects were re-activated. Renal projects were in the last tranche.  As such decisions had to be made as to which projects should be re-opened and refinanced. Unfortunately, the authors do not have access to the information that would be required to answer these questions raised by the reviewer.

**4. Report the data that were collected with greater granularity.** A short descriptive paragraph should report: number of the randomised patients within the sub-study (with arm allocation, if known), number of completed baseline ABPM, PWV, MoCA, BIA, and echocardiographic assessments, and explicit confirmation of the fate of the blood samples (destroyed on closure request). Even with numbers too small for inference, descriptive reporting strengthens the closure report's credibility.

 As written the funder required that all baseline samples and data were destroyed without analysis.

**5. Reconcile the sample-size figures.** The discrepancy between the 288 calculated in the manuscript and the 346 registered in the trial registry deserves one explanatory sentence in the *Sample size* section, most likely drop-out inflation, mid-study revision, or conservative registry registration.

 The higher number dates from the original grant application. The reviewers of the grant insisted on some changes in the investigational plan, as such the patient numbers were then recalculated based on the investigations requested by the reviewers.

**6. Correct editorial inconsistencies.** Unify the ethics code (18/EM/0213 vs 18/EM/0212), the second author's surname (McNeil vs MacNeill), correct "SITCHED" → "STITCHED" in the Discussion, add *N Engl J Med* to reference 24 (Blankestijn 2023, CONVINCE), and verify the Zenodo DOI (10.5281/zenodo18526386), which is currently non-functional and is self-cited as reference 23.

 This was corrected in a previous version but unfortunately the F1000 team could not post this into their web site

**7. Optional update on H4RT.** Given that H4RT is described as "in final analysis stage" and the OL-HDF landscape is moving quickly, a brief status update, or incorporation of any preliminary findings available by final submission will increase the manuscript's temporal relevance.

 Once these revisions have been implemented, the manuscript will be suitable for publication and will address a genuine unmet need within the field. Halted, partial, and null-result studies are infrequently disseminated in the nephrology literature, and this under-representation perpetuates publication

 This is not possible.

# Reviewer report: Reviewer response for version 1

**DOI:** 10.3310/nihropenres.15474.r40343

**Published:** 2026-05-11

**Authors:** Jakub Ruszkowski

**Affiliations:** 1 Medical University of Gdańsk, Gdańsk, Poland

The authors describe STITCHED, a mechanistic sub-study nested within the H4RT trial, designed to investigate why high-volume online haemodiafiltration (OL-HDF) might confer a survival advantage over high-flux haemodialysis. Owing to COVID-19 disruption and subsequent NIHR funding withdrawal, the study was halted before meaningful data could be collected. The manuscript functions as a hybrid protocol/closure report.

 This is a worthwhile contribution. The scientific rationale is genuinely strong and addresses a knowledge gap that neither H4RT nor the recently reported CONVINCE trial was designed to fill. Establishing why OL-HDF might improve survival has direct implications for dialyser design, dialysis machines, and dialysis-fluid composition — a positive outcome trial without mechanistic understanding leaves clinicians and manufacturers without the insight needed to refine treatment or develop simpler alternatives (such as the internal-convection dialysers the authors mention). Publishing a transparent account of an NIHR-funded study halted before completion is also valuable in itself.

 Comments:

 1. The article is published as a "Research Article" but contains no findings. As a loose suggestion, the authors might consider adjusting the title to signal the nature of the contribution — for example, "The STITCHED study: protocol and closure report of a mechanistic sub-study halted by COVID-19" — or adding a sentence to the Introduction making the genre explicit. Either would orient readers without requiring substantive rewriting.

 2. The authors make an important point in the Conclusion: "By comparing these changes in arterial stiffness, cardiac modelling, and cognitive function, STITCHED was designed to determine whether the improvement in survival was due to improved blood pressure control, removal of uraemic toxins, and changes in the thermal or sodium and calcium balance between diffusive and convective clearance." This is the central justification for the study, and the authors link each candidate mechanism well to a concrete consequence (dialyser design, machine architecture, fluid composition, blood-pressure targets). However, the listed primary and secondary objectives are framed exclusively as pairwise comparisons of individual parameters between HD and OL-HDF (intradialytic hypotension, BP variability, PWV, cognitive function, extracellular water, cardiac biomarkers, echocardiographic parameters, β2-microglobulin clearance), without any objective explicitly linking these intermediate measures to the survival outcome from H4RT — for example through formal mediation analysis treating dialysis modality as the exposure, the STITCHED parameters as candidate mediators, and survival or cardiovascular events as the outcome. As stated, the objectives would establish *that* the modalities differ on each measure but not adjudicate *which* of those differences accounts for the survival advantage, which is the question the narrative actually poses. It would help readers if the manuscript made the authors' position on this explicit — whether a pre-closure statistical analysis plan did include a mediation or related framework for relating the STITCHED measurements to H4RT outcomes (in which case a brief summary would be welcome), or whether STITCHED was conceived as a descriptive/hypothesis-generating study with formal mediation analyses envisaged for a follow-on study. Either framing is entirely defensible; clarifying which applies would resolve the tension and, in the latter case, sketching what such a successor study might look like would itself be a valuable contribution.

 3. Reflective perspective is underdeveloped. The genuinely instructive material — the pandemic disruption, staff attrition, the NIHR review process, the CRF subcontracting, and the funding withdrawal despite revised milestones being met — is currently confined to the Discussion. Expanding the "Reflective/critical perspective" section with what the authors would do differently and what protections mechanistic sub-studies need when nested within larger trials would be among the most original contributions of the paper.

 4. Inconsistencies/typographical errors should be corrected: the ethics committee reference differs between paragraphs (18/EM/0213 vs 18/EM/0212); the second author's surname appears as both "McNeil" (title and author list) and "MacNeill" (Competing Interests); and "SITCHED" appears in place of "STITCHED" in the Discussion.

 5. Reference 24 is missing the journal name.

 6. The Zenodo DOI (10.5281/zenodo18526386) does not work.

Is the work clearly and accurately presented and does it cite the current literature?

Partly

If applicable, is the statistical analysis and its interpretation appropriate?

Not applicable

Are all the source data underlying the results available to ensure full reproducibility?

No source data required

Is the study design appropriate and is the work technically sound?

Yes

Are the conclusions drawn adequately supported by the results?

Yes

Are sufficient details of methods and analysis provided to allow replication by others?

Yes

Reviewer Expertise:

Nephrology

I confirm that I have read this submission and believe that I have an appropriate level of expertise to confirm that it is of an acceptable scientific standard, however I have significant reservations, as outlined above.

# Response

**Published:** 2026-05-15

**Authors:** Andrew Davenport

Comments:

 1. The article is published as a "Research Article" but contains no findings. As a loose suggestion, the authors might consider adjusting the title to signal the nature of the contribution — for example, "The STITCHED study: protocol and closure report of a mechanistic sub-study halted by COVID-19" — or adding a sentence to the Introduction making the genre explicit. Either would orient readers without requiring substantive rewriting.

 Thank you for this suggestion to amend the title. Tin keeping with the NIHR regulations this is reported as a research article.

 2. The authors make an important point in the Conclusion: "By comparing these changes in arterial stiffness, cardiac modelling, and cognitive function, STITCHED was designed to determine whether the improvement in survival was due to improved blood pressure control, removal of uraemic toxins, and changes in the thermal or sodium and calcium balance between diffusive and convective clearance." This is the central justification for the study, and the authors link each candidate mechanism well to a concrete consequence (dialyser design, machine architecture, fluid composition, blood-pressure targets). However, the listed primary and secondary objectives are framed exclusively as pairwise comparisons of individual parameters between HD and OL-HDF (intradialytic hypotension, BP variability, PWV, cognitive function, extracellular water, cardiac biomarkers, echocardiographic parameters, β2-microglobulin clearance), without any objective explicitly linking these intermediate measures to the survival outcome from H4RT — for example through formal mediation analysis treating dialysis modality as the exposure, the STITCHED parameters as candidate mediators, and survival or cardiovascular events as the outcome. As stated, the objectives would establish *that* the modalities differ on each measure but not adjudicate *which* of those differences accounts for the survival advantage, which is the question the narrative actually poses. It would help readers if the manuscript made the authors' position on this explicit — whether a pre-closure statistical analysis plan did include a mediation or related framework for relating the STITCHED measurements to H4RT outcomes (in which case a brief summary would be welcome), or whether STITCHED was conceived as a descriptive/hypothesis-generating study with formal mediation analyses envisaged for a follow-on study. Either framing is entirely defensible; clarifying which applies would resolve the tension and, in the latter case, sketching what such a successor study might look like would itself be a valuable contribution.

 As highlighted by the reviewer’s comment the study was designed to provide guidance for a later study to concentrate on the most probable factors leading to improved patient survival.

 3. Reflective perspective is underdeveloped. The genuinely instructive material — the pandemic disruption, staff attrition, the NIHR review process, the CRF subcontracting, and the funding withdrawal despite revised milestones being met — is currently confined to the Discussion. Expanding the "Reflective/critical perspective" section with what the authors would do differently and what protections mechanistic sub-studies need when nested within larger trials would be among the most original contributions of the paper.

 This section was reviewed and edited by the NIHR editorial team

 4. Inconsistencies/typographical errors should be corrected: the ethics committee reference differs between paragraphs (18/EM/0213 vs 18/EM/0212); the second author's surname appears as both "McNeil" (title and author list) and "MacNeill" (Competing Interests); and "SITCHED" appears in place of "STITCHED" in the Discussion.

 Thank you, this has been amended

 5. Reference 24 is missing the journal name.

 Thank you, this has been amended

 6. The Zenodo DOI (10.5281/zenodo18526386) does not work.

 Thank you, this has been amended and reloaded as Doi 10.5281/zenodo.20154880

- Is the work clearly and accurately presented and does it cite the current literature?

Partly

 The literature referenced reflects that published at the time that the study was approved.

# Response

**Published:** 2026-05-29

**Authors:** Andrew Davenport

The authors describe STITCHED, a mechanistic sub-study nested within the H4RT trial, designed to investigate why high-volume online haemodiafiltration (OL-HDF) might confer a survival advantage over high-flux haemodialysis. Owing to COVID-19 disruption and subsequent NIHR funding withdrawal, the study was halted before meaningful data could be collected. The manuscript functions as a hybrid protocol/closure report.

 This is a worthwhile contribution. The scientific rationale is genuinely strong and addresses a knowledge gap that neither H4RT nor the recently reported CONVINCE trial was designed to fill. Establishing why OL-HDF might improve survival has direct implications for dialyser design, dialysis machines, and dialysis-fluid composition — a positive outcome trial without mechanistic understanding leaves clinicians and manufacturers without the insight needed to refine treatment or develop simpler alternatives (such as the internal-convection dialysers the authors mention). Publishing a transparent account of an NIHR-funded study halted before completion is also valuable in itself.

 Comments:

 1. The article is published as a "Research Article" but contains no findings. As a loose suggestion, the authors might consider adjusting the title to signal the nature of the contribution — for example, "The STITCHED study: protocol and closure report of a mechanistic sub-study halted by COVID-19" — or adding a sentence to the Introduction making the genre explicit. Either would orient readers without requiring substantive rewriting.

**Response**:Thank you for this suggestion to amend the title. In keeping with the NIHR regulations this is reported as a research article.

 2. The authors make an important point in the Conclusion: "By comparing these changes in arterial stiffness, cardiac modelling, and cognitive function, STITCHED was designed to determine whether the improvement in survival was due to improved blood pressure control, removal of uraemic toxins, and changes in the thermal or sodium and calcium balance between diffusive and convective clearance." This is the central justification for the study, and the authors link each candidate mechanism well to a concrete consequence (dialyser design, machine architecture, fluid composition, blood-pressure targets). However, the listed primary and secondary objectives are framed exclusively as pairwise comparisons of individual parameters between HD and OL-HDF (intradialytic hypotension, BP variability, PWV, cognitive function, extracellular water, cardiac biomarkers, echocardiographic parameters, β2-microglobulin clearance), without any objective explicitly linking these intermediate measures to the survival outcome from H4RT — for example through formal mediation analysis treating dialysis modality as the exposure, the STITCHED parameters as candidate mediators, and survival or cardiovascular events as the outcome. As stated, the objectives would establish *that* the modalities differ on each measure but not adjudicate *which* of those differences accounts for the survival advantage, which is the question the narrative actually poses. It would help readers if the manuscript made the authors' position on this explicit — whether a pre-closure statistical analysis plan did include a mediation or related framework for relating the STITCHED measurements to H4RT outcomes (in which case a brief summary would be welcome), or whether STITCHED was conceived as a descriptive/hypothesis-generating study with formal mediation analyses envisaged for a follow-on study. Either framing is entirely defensible; clarifying which applies would resolve the tension and, in the latter case, sketching what such a successor study might look like would itself be a valuable contribution.

**Response: **As highlighted by the reviewer’s comment the study was designed to provide guidance for a later study to concentrate on the most probable factors leading to improved patient survival.

 3. Reflective perspective is underdeveloped. The genuinely instructive material — the pandemic disruption, staff attrition, the NIHR review process, the CRF subcontracting, and the funding withdrawal despite revised milestones being met — is currently confined to the Discussion. Expanding the "Reflective/critical perspective" section with what the authors would do differently and what protections mechanistic sub-studies need when nested within larger trials would be among the most original contributions of the paper.

**Response: ** This section was reviewed and edited by the NIHR editorial team

 4. Inconsistencies/typographical errors should be corrected: the ethics committee reference differs between paragraphs (18/EM/0213 vs 18/EM/0212); the second author's surname appears as both "McNeil" (title and author list) and "MacNeill" (Competing Interests); and "SITCHED" appears in place of "STITCHED" in the Discussion.

**Response: ** Thank you, this has been amended

 5. Reference 24 is missing the journal name.

**Response: ** Thank you, this has been amended

 6. The Zenodo DOI (10.5281/zenodo18526386) does not work.

**Response: ** Thank you, this has been amended and reloaded as Doi 10.5281/zenodo.20154880

- Is the work clearly and accurately presented and does it cite the current literature?

Partly

 The literature referenced reflects that published at the time that the study was approved.

# Response

**Published:** 2026-06-16

**Authors:** Andrew Davenport

Comments:

 1. The article is published as a "Research Article" but contains no findings. As a loose suggestion, the authors might consider adjusting the title to signal the nature of the contribution — for example, "The STITCHED study: protocol and closure report of a mechanistic sub-study halted by COVID-19" — or adding a sentence to the Introduction making the genre explicit. Either would orient readers without requiring substantive rewriting.

***Thank you for this suggestion to amend the title. Tin keeping with the NIHR regulations this is reported as a research article.***

 2. The authors make an important point in the Conclusion: "By comparing these changes in arterial stiffness, cardiac modelling, and cognitive function, STITCHED was designed to determine whether the improvement in survival was due to improved blood pressure control, removal of uraemic toxins, and changes in the thermal or sodium and calcium balance between diffusive and convective clearance." This is the central justification for the study, and the authors link each candidate mechanism well to a concrete consequence (dialyser design, machine architecture, fluid composition, blood-pressure targets). However, the listed primary and secondary objectives are framed exclusively as pairwise comparisons of individual parameters between HD and OL-HDF (intradialytic hypotension, BP variability, PWV, cognitive function, extracellular water, cardiac biomarkers, echocardiographic parameters, β2-microglobulin clearance), without any objective explicitly linking these intermediate measures to the survival outcome from H4RT — for example through formal mediation analysis treating dialysis modality as the exposure, the STITCHED parameters as candidate mediators, and survival or cardiovascular events as the outcome. As stated, the objectives would establish *that* the modalities differ on each measure but not adjudicate *which* of those differences accounts for the survival advantage, which is the question the narrative actually poses. It would help readers if the manuscript made the authors' position on this explicit — whether a pre-closure statistical analysis plan did include a mediation or related framework for relating the STITCHED measurements to H4RT outcomes (in which case a brief summary would be welcome), or whether STITCHED was conceived as a descriptive/hypothesis-generating study with formal mediation analyses envisaged for a follow-on study. Either framing is entirely defensible; clarifying which applies would resolve the tension and, in the latter case, sketching what such a successor study might look like would itself be a valuable contribution.

***As highlighted by the reviewer’s comment the study was designed to provide guidance for a later study to concentrate on the most probable factors leading to improved patient survival.***

 3. Reflective perspective is underdeveloped. The genuinely instructive material — the pandemic disruption, staff attrition, the NIHR review process, the CRF subcontracting, and the funding withdrawal despite revised milestones being met — is currently confined to the Discussion. Expanding the "Reflective/critical perspective" section with what the authors would do differently and what protections mechanistic sub-studies need when nested within larger trials would be among the most original contributions of the paper.

***This section was reviewed and edited by the NIHR editorial team***

 4. Inconsistencies/typographical errors should be corrected: the ethics committee reference differs between paragraphs (18/EM/0213 vs 18/EM/0212); the second author's surname appears as both "McNeil" (title and author list) and "MacNeill" (Competing Interests); and "SITCHED" appears in place of "STITCHED" in the Discussion.

***Thank you, this has been amended***

 5. Reference 24 is missing the journal name.

***Thank you, this has been amended***

 6. The Zenodo DOI (10.5281/zenodo18526386) does not work.

***Thank you, this has been amended and reloaded as Doi 10.5281/zenodo.20154880***

- Is the work clearly and accurately presented and does it cite the current literature?

Partly

 The literature referenced reflects that published at the time that the study was approved.

# Reviewer report: Reviewer response for version 1

**DOI:** 10.3310/nihropenres.15474.r40345

**Published:** 2026-05-04

**Authors:** Jeroen Vollenbroek, Robin Vernooij

**Affiliations:** 1 Nephrology, University Medical Centre Utrecht, Utrecht, The Netherlands

The manuscript describes a clinical study aimed at looking into the cause of improved dialysis outcome with hemodiafiltration over high flux hemodialysis with a hypotehsized cause being the better blood pressure regulation during HDF with far fewer drops in blood pressure. 

 The rationale is strong and clinically relevant, as existing outcome trials have provided limited insight into the underlying pathways. Overall, the methodology and proposed measurements are appropriate and well described.

 However, the main issue is that the manuscript does not clearly state what type of publication this is intended to be. It reads largely as a protocol or methods paper, but no results are presented. The authors should clarify this explicitly in the Introduction.

 The authors should also state whether any patient data were collected, even if minimal. For example, were any patients randomized into this substudy? Were any baseline or follow-up measurements performed? If 98 patients consented but no meaningful measurements were obtained, this should be clearly reported.

 In addition, the authors should explain whether the study will still be performed, whether any follow-up analysis is planned, or whether the substudy has been discontinued. If the study could not be completed, the reasons should be briefly described.

 There also appears to be a small inconsistency between the manuscript sample size of 288 and the target sample size registered for the trial, which is 346. The authors should reconcile or explain this discrepancy.

 In summary, the study concept is valuable and the methodology appears sound, but the manuscript needs clearer framing, transparency about available data and study status, and an explanation of its added value in the absence of results.

Is the work clearly and accurately presented and does it cite the current literature?

No

If applicable, is the statistical analysis and its interpretation appropriate?

Not applicable

Are all the source data underlying the results available to ensure full reproducibility?

No

Is the study design appropriate and is the work technically sound?

Yes

Are the conclusions drawn adequately supported by the results?

No

Are sufficient details of methods and analysis provided to allow replication by others?

Partly

Reviewer Expertise:

Dialysis innovation, Dialysate regeneration, artificial organs

We confirm that we have read this submission and believe that we have an appropriate level of expertise to confirm that it is of an acceptable scientific standard, however we have significant reservations, as outlined above.

# Response

**Published:** 2026-06-16

**Authors:** Andrew Davenport

However, the main issue is that the manuscript does not clearly state what type of publication this is intended to be. It reads largely as a protocol or methods paper, but no results are presented. The authors should clarify this explicitly in the Introduction.

*The article has been submitted and written according to the NIHR guidelines. We have amended the introduction to aid clarity.*

 The authors should also state whether any patient data were collected, even if minimal. For example, were any patients randomized into this substudy? Were any baseline or follow-up measurements performed? If 98 patients consented but no meaningful measurements were obtained, this should be clearly reported.

*It is stated that all biological samples were destroyed before any analysis. Patient data was collected as part of the larger H4RT study. Patients had been randomised when the enrolled into the H4RT study, but there was no randomisation for STITCHED as this was planned as an observational study.*

 In addition, the authors should explain whether the study will still be performed, whether any follow-up analysis is planned, or whether the substudy has been discontinued. If the study could not be completed, the reasons should be briefly described.

*As stated the funding body closed down the study. The NIHR team edited the paper.*

 There also appears to be a small inconsistency between the manuscript sample size of 288 and the target sample size registered for the trial, which is 346. The authors should reconcile or explain this discrepancy.

*There was a recalculation of the numbers required from the original estimation.*

 In summary, the study concept is valuable and the methodology appears sound, but the manuscript needs clearer framing, transparency about available data and study status, and an explanation of its added value in the absence of results.

- Is the work clearly and accurately presented and does it cite the current literature?

The work has to be considered in the context of what was known at the time of the study design.

- Is the study design appropriate and is the work technically sound?

Yes

- Are sufficient details of methods and analysis provided to allow replication by others?
- Partly

  As the study was halted it is not possible to comment

- If applicable, is the statistical analysis and its interpretation appropriate?

Not applicable

- Are all the source data underlying the results available to ensure full reproducibility?

No

 As the study was halted prior to collecting any results, then by definition there is no data available.

- Are the conclusions drawn adequately supported by the results?

No

 As the study was halted prior to collecting any results, then by definition any conclusion cannot be supported by the results.
